# Regional heterogeneity of striatal cholinergic interneurons: setting the stage for diverse behavioral repertoires

**DOI:** 10.3389/fncel.2026.1718947

**Published:** 2026-02-24

**Authors:** Julia C. Lemos, Mariana Duhne, Nao Chuhma

**Affiliations:** 1 Department of Neuroscience, University of Minnesota, Minneapolis, MN, United States; 2 Medical Discovery Team on Addiction, University of Minnesota, Minneapolis, MN, United States; 3 Department of Neurology, University of California, San Francisco, San Francisco, CA, United States; 4 Department of Molecular Therapeutics, New York State Psychiatric Institute, New York, NY, United States; 5 Department of Psychiatry, Columbia University, New York, NY, United States

**Keywords:** acetylcholine, cholinergic interneuron, dopamine, nucleus accumbens, striatum

## Abstract

Cholinergic interneurons (ChIs) constitute only a small fraction of striatal neurons, yet their dense axonal arborizations and widespread acetylcholine release make them powerful regulators of basal ganglia output. While the striatum is often described as cytoarchitecturally uniform, accumulating evidence reveals significant regional heterogeneity in ChI properties that shape striatal computation and behavior. Recent observations suggest that ChI heterogeneity occurs at multiple levels, extending beyond simple dorsal–ventral differences. This includes morphological, electrophysiological, and molecular heterogeneity among ChIs, as well as ChI-driven behaviors in the dorsolateral striatum, dorsomedial striatum, and nucleus accumbens (core and shell). Despite these accumulating observations, most studies and reviews of ChIs focus narrowly on one or two functional levels. As a result, a systematic and comprehensive comparison of ChI activity and its modulation across finer striatal subregions and multiple levels of analysis has not been undertaken. Here, we integrate findings across cellular, circuit, and behavioral levels to frame how regional ChI heterogeneity may set the stage for diverse behavioral repertoires. In light of recent studies, we highlight ChI activity and dopamine neuron–ChI interactions and compare mechanisms of cholinergic modulation across striatal subregions. This integrative perspective reveals critical discrepancies in the current literature, which should be addressed experimentally to understand how ChIs contribute to regionally distinct behaviors in both healthy and pathological states.

## Introduction

The basal ganglia is a series of interconnected nuclei that transform activity in the cortex to control movement, motivation and reward processing (Gerfen and Bolam, 2016; Macpherson and Hikida, 2019). The striatum is the major input structure of the basal ganglia, receiving inputs from all regions of the cortex and processing the information by integrating glutamatergic inputs from the thalamus and limbic system, GABAergic inputs from the pallidum and ventral midbrain, monoaminergic inputs from the midbrain, and peptidergic inputs as co-transmission (Graybiel and Mink, 2009; Pennartz et al., 2009). In human and non-human primates, the striatum is comprised of the caudate, putamen, and nucleus accumbens (NAc) which is further divided into shell and core. In rodents, there is no clear separation between the caudate and putamen, although the dorsolateral and dorsomedial striatum are homologous to the caudate and putamen, respectively. (Hilario and Costa, 2008; Hunnicutt et al., 2016; Macpherson and Hikida, 2019; Scofield et al., 2016).

Box 1 **Conceptual principles emerging from regional heterogeneity of striatal cholinergic interneurons.**

Although numerous studies have documented regional heterogeneity in striatal cholinergic interneuron (ChI) properties, these findings span different experimental levels (*cellular*, *synaptic*, circuit, and behavioral), making it difficult to extract unifying principles. Below, we synthesize current evidence to bring forth key conceptual conclusions that emerge across levels of analysis.

- **ChI heterogeneity operates at a finer spatial scale than canonical striatal subdivisions.**

Differences in intrinsic firing properties, synaptic modulation, and cell density are observed not only between dorsal and ventral striatum, or core and shell, but also within subregions, including localized microdomains or “hot spots.” Apparent discrepancies across studies may reflect differences in recording location rather than experimental inconsistency.

- **Intrinsic ChI properties constrain, but do not dictate, *in vivo* activity.**

*Ex vivo* measurements define a cell’s intrinsic firing “default mode,” yet *in vivo* firing emerges from continuous integration of dynamic synaptic inputs and behavioral contexts. Divergence between *ex vivo* and *in vivo* observations therefore reflects biological context rather than technical validity.

- **The nature of dopamine–ChI interactions is regionally heterogeneous and multimodal.**

Midbrain dopamine neurons influence ChIs through distinct combinations of dopamine, glutamate, and GABA co-transmission across striatal subregions, producing diverse firing motifs rather than a uniform triphasic response. Canonical burst–pause–rebound dynamics are thus not obligatory or globally conserved.

- **ChI-driven modulation of dopamine release reflects temporally precise, microdomain-specific signaling.**

nAChR- and mAChR-dependent dopamine transmission is robust in *ex vivo* preparations and varies markedly across striatal subregions and microdomains. *In vivo* detection likely depends on precise sampling of local microcircuits, behavioral synchronization of ChI activity, and sufficient temporal resolution to capture fast cholinergic–dopaminergic interactions, suggesting that some negative findings reflect undersampling rather than absence of local control.

- **Regional ChI properties differentially affect striatal circuits driving distinct behaviors.**

Regional differences in ChI firing, modulation, and cholinergic–dopaminergic modify striatal subregions in concert and drive specific modes of learning, motivation, and behavioral flexibility.

These striatal subregions show remarkably similar cytoarchitecture. The striatum is comprised primarily of GABAergic medium spiny projection neurons (SPNs) (~95%) divided into two subtypes of direct pathway SPNs and indirect pathway SPNs, which show distinct projection patterns, dopamine receptor expression, electrophysiological properties, and peptidergic co-transmitters (Castro and Bruchas, 2019; Gerfen, 2022; Tepper and Bolam, 2004). Direct pathway SPNs express dopamine D1 receptors and send axons directly to output nuclei of basal ganglia, i.e., the substantia nigra (SN), ventral tegmental area (VTA), and internal segment of globus pallidus (entopeduncular nucleus in rodents), while indirect pathway SPNs express dopamine D2 receptors and output through the external segment of globus pallidus or ventral pallidum. Note that a recognizable fraction of direct pathway SPNs both in rodents and primates send collateral projections to external segment of the globus pallidus (bridging collaterals) or projections to the ventral pallidum (Cazorla et al., 2014; Fujiyama et al., 2011; Levesque and Parent, 2005; Smith et al., 2013). The rest of striatal neurons are GABAergic interneurons (~2–3% of neurons) and cholinergic interneurons (ChIs) (1–2%), which are the primary source of acetylcholine (ACh) in the striatum (Castro and Bruchas, 2019; Gerfen, 2022). While ChIs are only a small fraction of the striatal neurons, they influence large number of SPNs with their high degree of ramification (Ratna and Francis, 2025), making them suitable to serve as “gate keepers” of striatal outputs.

The striatum receives rich innervation from midbrain dopamine neurons of the SN *pars compacta* (SNc) and VTA (Scofield et al., 2016). Dopamine exerts control of direct and indirect pathway SPN excitability via G_olf_-coupled D1-like receptors and G_i/o_-coupled D2-like receptors, respectively, (Calabresi et al., 2007; Surmeier et al., 2007), in addition to controlling ChI excitability through both D1- and D2-like receptors. Dopamine release in the striatum can be driven through canonical propagation of action potentials from soma but also can be triggered through presynaptic ACh receptors within the local microcircuit as well (Calabresi et al., 2006; Calabresi et al., 2007; Surmeier et al., 2017). The interaction between dopamine and ACh systems and its perturbation in the striatum has been recognized as a critical mechanism in pathogenesis of neurological disorders. Interest in this interaction has re-emerged with findings that local reciprocal synaptic ACh-dopamine interactions are a novel underpinning mechanism of learning and decision-making (Kim et al., 2019).

Despite a very similar cytoarchitecture across the entire striatum, each subregion of the striatum mediates different behaviors (Hilario and Costa, 2008; Hunnicutt et al., 2016; Macpherson and Hikida, 2019; Obeso et al., 2014). For example, the caudate or dorsolateral striatum (DLS) mediates motor planning, motor coordination and stimulus–response learning, while the dorsomedial striatum (DMS) mediates goal-directed or response-outcome learning and is critical in decision making (Hilario and Costa, 2008). The NAc core and shell play critical roles in reinforcement learning and reward valuation (Chen et al., 2021; Macpherson and Hikida, 2019). Although these subregional differences in mediating behaviors are often attributed to different sources of cortical and subcortical glutamatergic inputs, those are not the sole determinants. Meso-striatal dopamine projections are arranged topographically, and show heterogeneity in its transmission, its modulation, and in its mediation of behaviors that depends on target subregions (Haber and Behrens, 2014; Ikemoto, 2007). Likewise, ChIs also show regional heterogeneity in intrinsic excitability, their modulation, and local circuit control. As both dopamine projections and ChIs are capable of exerting strong control over a large number of SPNs, meso-striatal dopamine projection and ChI heterogeneity may play a critical role in determining regionally distinct behaviors as well.

In this review, we feature the regional heterogeneity of reciprocal interactions between ChIs and dopamine neurons at the cellular and microcircuit level, highlighting recent studies in which fine regional analyses have been performed. We extend this discussion to the systems level with the aim of revealing potential relevance for shaping regionally distinct behavioral repertoires. Recent advances in genetic targeting and recording now allow identified neurons of distinct classes to be monitored *in vivo*, alongside measurements of local dopamine release. In parallel, increasingly precise tools enable manipulation of neuronal firing with high specificity and temporal resolution to investigate these interactions, providing new opportunities to link ChI activity to behavior across striatal subregions. At the same time, while *in vivo* studies often prioritize behavioral and circuit-level questions, fine-scale regional heterogeneity revealed *ex vivo* is not always incorporated into experimental design or interpretation. Integrating findings across these levels therefore remains an ongoing challenge. By laying out and comparing current studies of ChIs across experimental approaches, this review aims to clarify points of convergence, elucidate potential explanations for discrepancies, identify knowledge gaps, and guide future directions.

## Regional heterogeneity in cellular properties

### ChI morphology

ChIs have substantially larger soma size compared to other striatal neuron types and 2–4 large primary dendrites that bifurcate repeatedly but infrequently (Goldberg and Wilson, 2016; Gonzales and Smith, 2015; Kawaguchi et al., 1995; Poppi et al., 2021; Zhou et al., 2002). Although ChIs are regarded as aspiny neurons, thin spine-like structures are observed sparsely (Phelps et al., 1985; Poppi et al., 2021). The ChI dendritic field is widespread, subtending a much larger region than projection neurons, indicating that ChIs converge input signals within a large area (Gonzales and Smith, 2015; Kawaguchi et al., 1995). Likewise, ChIs have a dense and large axonal field, which is well suited for strong control of local striatal circuitry (Goldberg and Wilson, 2016; Zhou et al., 2002). The ChI axon arbor is one of the densest ACh arbors in the brain, and each ChI axon arbor has about 500,000 varicosities in rat neostriatum (Gonzales and Smith, 2015; Zhou et al., 2002). About 90% of these varicosities are not synaptic, suggesting that ACh works through volume transmission (Contant et al., 1996; Descarries et al., 1997; Nosaka and Wickens, 2022), in addition to fast synaptic transmission (Abudukeyoumu et al., 2019; Assous et al., 2017; English et al., 2011; Faust et al., 2015; Threlfell et al., 2012).

Although these morphological properties are quite similar across the striatal subregions, ChIs in the NAc tend to have smaller soma and fewer dendritic branches than those in the dorsal striatum (DS) (Gonzales and Smith, 2015; Phelps and Vaughn, 1986; Ztaou et al., 2021; Table 1). It is not clear whether size of ChI soma affects functionality, however, size of dendritic field may affect excitability. In SPNs, computational simulation suggests that shorter total length and fewer branching of dendrites cause higher excitability (Gertler et al., 2008). This implies that NAc ChIs might be more excitable than those in the DS, which indeed appears to be the case in *ex vivo* preparations (see below). However, there is a subpopulation of ChIs in the DLS co-transmitting GABA, which has slower firing frequency, more voltage sag at hyperpolarizing current injection, and smaller dendritic field (Lozovaya et al., 2018), refuting the hypothesis that ChIs with smaller dendritic field could be more excitable, as observed in SPNs.

**Table 1:** Regional heterogeneity in intrinsic cholinergic interneuron properties and reciprocal local regulation between dopamine neurons and cholinergic interneurons.

Category	Measurement	DS		VS (NAc)		References
		Lateral	Medial	Core	Shell	
Morphology	Soma size	++		+		Gonzales and Smith (2015), Phelps and Vaughn (1986), and Ztaou et al. (2021)
	Dendritic branches	++		+		Gonzales and Smith (2015) and Phelps and Vaughn (1986)
	Density	++	++++	+	++	Adrover et al. (2020), Gonzales and Smith (2015), Lemos et al. (2019), Matamales et al. (2016), and Olson et al. (2024)
Firing properties	Firing rate (*ex vivo*)	++	+	++	+++	Bennett and Wilson (1999), Chuhma and Rayport (2024), and Lemos et al. (2019)
	Firing rate (*in vivo*)	+++	++	+		Adler et al. (2013) and Duhne et al. (2024)
	Pause onset timing	later	earlier			Aosaki et al. (1995)
DA release modulation by ACh	nAChR (*ex vivo*)	+	+	+	+++	Cachope et al. (2012), Threlfell et al. (2012), Lemos et al. (2019), Adrover et al. (2020), Shin et al. (2015), Kramer et al. (2022), Liu et al. (2022), and Shin et al. (2017)
	nAChR (*in vivo*)	+/− (mixed findings)	+/− (mixed findings)	++		Toth et al. (1992), Nakamura et al. (1992), Chantranupong et al. (2023), Krok et al. (2023), Mohebi et al. (2023), Duhne et al. (2024), Costa et al. (2025), Touponse et al. (2026), Flink et al. (2025), and Goldbach et al. (2025)
	mAChR, non-specified (*in vivo*)	+				Flink et al. (2025)
	M2R/M4R (*ex vivo*)	+	+	+	++	Threlfell et al. (2010)
	M5R (*in vivo*/*ex vivo*)			++	++	Shin et al. (2015) and Razidlo et al. (2022)
DA neuron synapse	D2R (*ex vivo*/*in vivo*)	+++	++++	++	+	Chuhma et al. (2014), Cai and Ford (2018), Gallo et al. (2022), and Chuhma et al. (2023)
	D1/5R (*ex vivo*)	+			+ (OT)	Chuhma et al. (2018), Chuhma et al. (2023), and Wieland et al. (2014)
	Glutamate (*ex vivo*)	+ (anterior)			+ (medial)	Cai and Ford (2018), Chuhma et al. (2018), and Chuhma et al. (2023)

Number of + indicates strength of the responses or modulation. In Morphology section, number of + indicates robustness of the observation, i.e., larger soma size, more dendritic branches, and higher density. DS, dorsal striatum; VS, ventral striatum; NAc, nucleus accumbens; OT, olfactory tubercle; DA, dopamine. Note that a sample of only selected references are shown for dopamine release modification by nAChR.

### ChI anatomical distribution

The striatum has two compartments, striosomes and matrix, distinguished by expression of transmitter-related signaling molecules (e.g., mu opioid receptor expression) and input–output connections (Crittenden and Graybiel, 2011). ChIs are frequently located at the borders between these two compartments, known as the peristriosomal zone (Brimblecombe and Cragg, 2017; Crittenden et al., 2014). ChI distribution within the striatum shows regional heterogeneity with higher density in the DS and NAc shell, and with the lowest density in the NAc core (Gonzales and Smith, 2015; Lemos et al., 2019; Matamales et al., 2016; Olson et al., 2024; Table 1). Although the low density of ChIs in the NAc core is conserved across rodents and primates, ChI density difference within the DS show some discrepancies. Some studies reported no significant difference between the DLS and DMS (Lemos et al., 2019; Olson et al., 2024), while others report significant difference in ChI densities between posterior DLS and posterior DMS (Matamales et al., 2016) or among three subregions along with dorsoventral axis in sagittal sections (Adrover et al., 2020). These discrepancies could be due to difference in detection methods, e.g., *in situ* hybridization to choline acetyltransferase (ChAT) mRNAs, immunoreactivity (ir) to ChAT protein, or transgenic fluorescent tagging driven by ChAT promotor, or due to different subregion delineation, e.g., to what extent the caudal aspect or “tail” of the striatum is included. In addition to density differences between the shell and core, ChAT-ir reveals a finer difference of ChI density within the NAc; a higher density of ChIs in the medial NAc compared to more lateral part of the NAc (Meredith et al., 1989; Phelps and Vaughn, 1986). There are sex-differences in ChI density across different subregions of the striatum; ChIs identified by fluorescent *in situ* hybridization to *Chat* mRNA show higher density in females in both the dorsal striatum and the NAc (Olson et al., 2024). However, if ChIs are identified by ChAT-ir, females show either lower or no difference in ChI density in the DS or NAc (Olson et al., 2024; Van Zandt et al., 2024). This discrepancy might be due to higher expression but lower translation efficacy of *Chat* mRNA in females.

Throughout the striatum, ChIs form clusters, (Gangarossa et al., 2013; Matamales et al., 2016), which are particularly robust in the anterior DS. While functional relevance of these clusters are not totally understood, one possibility is that these clusters might determine synchronized activity of multiple ChIs through local synaptic control (Dorst et al., 2020).

### Regional heterogeneity of ChI firing properties and ionic conductances

ChIs are electrophysiologically characterized by tonic spontaneous firing, both *in vivo* and *ex vivo*, shallow resting membrane potentials (~ − 60 mV), and voltage sag in response to negative step current injection due to delayed activation of hyperpolarization-activated cyclic nucleotide-gated cation (HCN) channels (Bennett and Wilson, 1999; Chuhma et al., 2014; Goldberg and Wilson, 2016; Kawaguchi, 1993; Kreitzer, 2009; Wilson, 2005; Wilson et al., 1990). Canonically, ChIs have wider action potentials compared to other types of neurons in the striatum (Chuhma and Rayport, 2024; Goldberg and Wilson, 2005; Kawaguchi, 1993). ChIs have large cell capacitance, mostly determined by distal dendrites (Hjorth et al., 2020). Together with higher input impedance (Chuhma and Rayport, 2024; Kawaguchi, 1993; Wilson et al., 1990), ChIs have slower membrane time constant (Chuhma and Rayport, 2024; Wilson et al., 1990) indicating slower voltage change in response to synaptic currents.

**Figure 1:**
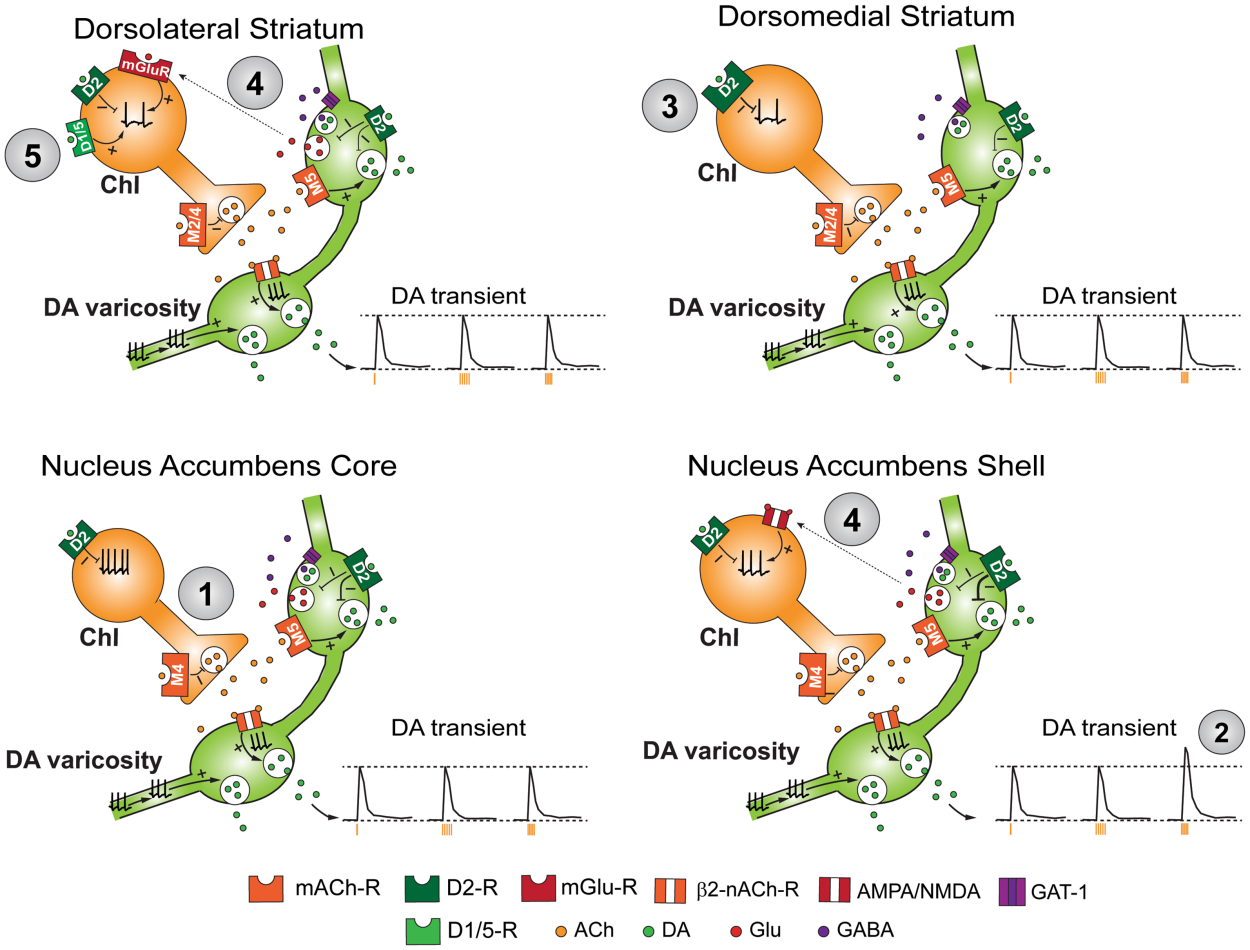
Schematic diagram summarizing heterogeneity in cellular properties of ChIs across striatal subregions. Summary of the regional differences in ChI/dopamine reciprocal modulation. Difference in intrinsic excitability recorded *ex vivo* is indicated by number of action potentials in axon soma (1). Dopamine transients triggered by single ChI photostimulation (amber tick; left trace), lower frequency train stimulation, e.g., 5 Hz (middle), and higher frequency train stimulation, e.g., 10 Hz are shown in insets (2). Dotted lines at bottom of traces indicate baseline level, and dotted lines at top of traces indicate dopamine transient level evoked by soma a single ChI stimulation. Dopamine D2R mediating inhibition of ChI firing (3) and the differential impact of glutamatergic co-transmission on ChI firing from dopamine varicosities across subregions (4). D1-like regulation of ChIs is predominantly seen in the DLS (5).

In slices, frequency of ChI spontaneous firing varies among subregions. Generally, NAc ChIs (3–15 Hz) show faster firing than DS ChIs (1–10 Hz). Among subregions, NAc shell ChIs show the fastest firing (3–15 Hz), while the slowest firing is observed in the medial DS (1–7 Hz) (Bennett and Wilson, 1999; Chuhma and Rayport, 2024; Lemos et al., 2019; Figure 1; Table 1).

Spontaneous firing of ChIs is caused by intrinsic voltage oscillation (Wilson, 2005) generated by orchestration of multiple types of ion channels; mostly determined by HCN currents and inward-rectifier K^+^ channels (Kir) (Codianni and Rubin, 2023; Goldberg and Wilson, 2016). A spontaneous single firing cycle is initiated by depolarization through HCN channels and persistent Na^+^ channels (Nav1.6). Action potentials generated by the depolarization causes Ca^2+^ influx through Q-type voltage-gated Ca^2+^ channels (Cav2.1), and this Ca^2+^ activates large conductance Ca^2+^-activated K^+^ channels (BK), which determines the falling phase of action potentials along with delayed-rectifier K^+^ channels. The action potential also activates N-type Ca^2+^ channels (Cav2.2) which activate small conductance Ca^2+^-activated K^+^ channels (SK) inducing after hyperpolarization (AHP). This AHP reprimes fast inactivating voltage-gated K^+^ channels (A-current; Kv4.2), making subthreshold depolarization slower for maintaining tonic repeated firing (Bennett et al., 2000; Goldberg and Wilson, 2016; Goldberg and Wilson, 2005; Maurice et al., 2004; Ratna and Francis, 2025; Song et al., 1998; Wilson, 2005). In contrast, L-type Ca^2+^ channels (principally Cav1.3 in neurons) provide the Ca^2+^ for a slow AHP that promotes rhythmic burst–pause transitions; blocking Cav2.2/SK favors bursting, while blocking Cav1/slow AHP suppresses it (Goldberg and Wilson, 2005).

Spontaneous firing, voltage sag at negative current injections, higher input impedance and slower membrane time constant, and shallow resting membrane potential are observed in ChIs in all striatal regions, however, those properties show regional difference. Under blockade of cholinergic receptors to avoid local cholinergic feedback, NAc shell ChIs have higher input impedance, less voltage sag, higher spontaneous firing rate, and steeper increase of firing in response to step current injection, compared to the DS (Chuhma and Rayport, 2024). When multiple properties are considered simultaneously (multivariate analysis), NAc shell ChIs are outstandingly different from all DS subregions and NAc core (Chuhma and Rayport, 2024). These observations suggest that ChIs in the NAc shell are easier to excite and steeply respond to increase of excitatory synaptic inputs compared to other striatal regions. Higher firing rate and higher density of ChIs (Matamales et al., 2016), imply higher baseline cholinergic tone in the NAc shell. Although attributing difference of intrinsic properties to expression difference of particular types of ion channels is very difficult, we can speculate some ion channels expressed heterogeneously (e.g., weaker expression of HCN channels judging from less voltage sag in NAc shell ChIs). While gene expression, including expression of ion channels, in ChIs were studied (Hjorth et al., 2020; Munoz-Manchado et al., 2018; Oyrer et al., 2019), regional difference within the striatum has not been systematically examined yet.

Individual ChIs show diverse firing patterns, and distribution of the individual ChI firing patterns is different among striatal subregions. In the DS, Bennett and colleagues identified three firing patterns: “regular,” “irregular,” and “rhythmic bursting,” based on firing rates and coefficients of variation of the inter-event interval distribution (Bennett and Wilson, 1999). In the NAc, ChIs exhibit four firing patterns: “regular,” “irregular,” “rhythmic bursting”—the same three modes as in the DS—and a “mixed mode” that has burst firing overlaid on regular tonic firing (Ingebretson et al., 2024). NAc core and shell ChIs exhibit different firing pattern distributions; in male mice, 80% of core ChIs show “regular” firing pattern, while only ~30% of shell ChIs show “regular” firing pattern (Ingebretson et al., 2024). The firing pattern distribution is plastic, affected by stress and estrous cycle. In the NAc core, but not in the NAc shell, repeated swim stress shifts ChI firing pattern distribution from mostly “regular” firing to irregular firing modes without changing overall firing rate after 7 days from swim session, while this change is restored to “regular” firing mode after 14 days post-stressor exposure (Ingebretson et al., 2024). In the NAc shell in females, ChI firing pattern varies across different periods of the estrous cycle (Ingebretson et al., 2024). NAc shell ChIs in females show generally lower firing frequency than in males; this is due to significantly low firing frequency in estrus period.

## Heterogeneity of firing patterns of cholinergic interneurons *in vivo*

For many years, tonically active neurons (TANs) have been regarded as putative ChIs *in vivo*, because definitive identification with genetic or biochemical methods was difficult. TANs are identified by electrophysiological properties, namely, firing rate of 3–15 Hz, wider spikes, tonic activity with long inter spike intervals, although the definition varies among studies (Aosaki et al., 1994a; Benhamou et al., 2014; Yamada et al., 2004). Traditionally, identification of ChIs *in vivo* is done by juxtacellular labeling with post-recording ChAT immunostaining (Inokawa et al., 2010; Sharott et al., 2012). The advent of optogenetics allowed for genetic identification of ChIs *in vivo* during recording in rodents (Atallah et al., 2014; Duhne et al., 2024), and potentially in primates as well (Hunker et al., 2025), which historically was not possible. “TAN” criteria have largely matched findings in optogenetically identified ChIs, however, there have been instances where optogenetically identified ChIs do not strictly adhere to “TAN” criteria, corroborating the electrophysiological diversity of these neurons.

Most *in vivo* studies have concentrated on recording from different regions within the DS as technical limitations have made it difficult to distinguish different subregions of the NAc. As far as we are aware, there are only two studies that have compared identified ChI action potential firing in multiple striatal subregions including the ventral striatum (VS) *in vivo*, and both of the studies found that ChIs recorded in the VS had lower average firing rates than in the DS (Adler et al., 2013; Duhne et al., 2024). These findings are not expected from *ex vivo* observations. Since these studies did not distinguish the NAc core and shell, the discrepancy could be due to differences in recording locations. However, these differences are also likely due to different firing control *in vivo* and *ex vivo*. The firing measured *ex vivo* is largely driven by cell intrinsic properties without effects of synaptic inputs driven by environmental context or behavioral repertoire (the cell’s “default mode.”) *In vivo* ChI firing is determined by balance of tone of multiple neurotransmitters in addition to intrinsic firing nature. For example, basal ChI activity *in vivo* is affected by tonic activity of ventral midbrain dopamine neurons (Grace and Onn, 1989; Paladini and Roeper, 2014). ChIs are influenced by continuous activity of cortical/subcortical glutamatergic and GABAergic inputs as well. Lower average basal firing rate in the NAc *in vivo* suggests that the NAc may have stronger inhibitory synaptic “tone” than the DS. Future studies of the balance between intrinsic properties of ChI, their inputs throughout the striatum, and their relevance during behavior is needed. The use of computational modeling and simulation can elucidate how differences in input strength converge on ChI’s with different “default modes” to produce *in vivo* firing patterns.

Firing of ChIs is also affected by behavioral states and environmental inputs, e.g., differential engagement of sensory inputs, reward presentation, and movement onsets (Benhamou et al., 2014; Bouabid et al., 2025; Yamada et al., 2004). For detailed discussion about ChI firing and behavioral state, see below (*Heterogeneity in ChI responses during behavioral tasks across striatal*).

TANs exhibit characteristic burst-pause-rebound responses to salient stimuli (Aosaki et al., 1994a; Kimura et al., 1984; Morris et al., 2004). Cortical and thalamic inputs evoke distinct patterns as well: triphasic responses by cortical inputs, simple pauses by thalamic inputs (Doig et al., 2014). The “pause” has been shown to be produced by a long after hyperpolarization (Reynolds et al., 2004), withdrawal of excitatory input (Zucca et al., 2018), D2 receptor-mediated inhibition (Chantranupong et al., 2023; Zucca et al., 2018), or inhibition from GABAergic interneurons or long-range GABA neurons (Al-Hasani et al., 2021; Brown et al., 2012; Dorst et al., 2020) (see below). It is likely that all these mechanisms contribute depending on behavioral contexts. However, little work has been done to elucidate the mechanisms. Future experiments should consider addressing the “source” of the pause in distinct behavioral contexts and the implications of these mechanisms for behavior. Regardless of the mechanisms, pause timing shows regional heterogeneity, e.g., pauses occur earlier in the caudate than the putamen (Aosaki et al., 1995). In the DS, the initial depolarization appears to be due to thalamic input (Matsumoto et al., 2001); however, functional thalamic connection is observed only in the DMS (Bradfield et al., 2013), suggesting mechanisms under initial depolarization may differ among striatal subregions.

## Regional heterogeneity in cellular modulation of ChIs: spotlight on reciprocal cholinergic-dopaminergic microcircuit interactions

Throughout the striatum there is a reciprocal relationship between ChIs and dopamine projections from the midbrain. Selective stimulation of dopamine terminals in the striatum affects ChI firing in a region specific way (Cai and Ford, 2018; Chuhma et al., 2023). Single pulse activation of ChIs can trigger action potentials in dopamine neuron *en passant* varicosities via β2-containing nicotinic ACh receptors (nAChRs) (Cachope et al., 2012; Kramer et al., 2022; Liu et al., 2022; Threlfell and Cragg, 2011; Threlfell et al., 2012). This interaction between ChIs and dopamine terminals has been observed *ex vivo* and *in vivo* in rodents and non-human primates (Cachope et al., 2012; Salinas et al., 2021; Shin et al., 2025; Threlfell et al., 2012), though there are some variations in strength of the effect across species.

### Cholinergic regulation of dopamine transmission

When dopamine release is measured using fast scan cyclic voltammetry (FSCV) in acute brain slices, dopamine transients evoked by a single electrical stimulus are reduced approximately to half with nAChR blockade (Lemos et al., 2019; Shin et al., 2017; Shin et al., 2015; Zhou et al., 2001). In contrast, dopamine transients evoked by selective stimulation of DA terminals via single pulse optogenetic stimulation are insensitive to nAChR blockade. These observations suggest that non-selective stimulation activates ChIs as well as dopamine terminals, and ACh released from ChIs enhances dopamine release through presynaptic nAChRs. Indeed, single pulse optogenetic stimulation of ChIs is sufficient to cause dopamine release, which is completely ablated by nAChR blockade (Adrover et al., 2020; Shin et al., 2017; Shin et al., 2015). Dopamine release recorded by amperometry coupled with photostimulation of either prefrontal cortex (PFC) projections to the striatum (see below), ChIs, or dopamine neuron terminals reveals the increasingly longer latency reflecting trisynaptic, disynaptic and direct evoking of dopamine release, respectively (Shin et al., 2015). These differences are smaller than sampling interval of FSCV (10 Hz), rendering them unresolvable by standard FSCV techniques. Perforated patch recordings from dopamine varicosities within the striatum demonstrated nAChR-dependent actional potential driven dopamine release locally with millisecond-order latency (Kramer et al., 2022; Kramer et al., 2025; Liu et al., 2022). These observations suggest that high temporal resolution recording is required *in vivo* to observe nAChR-induced DA release, which might be difficult with current tools. Dopamine release modulation through nAChR can be activated via astrocytes as well. Striatal astrocytes make “soma-to-soma” satellite contacts with ChIs, enabling subsecond modulation of ChI excitability via transient decreases in extracellular calcium, which affects dopamine release through presynaptic nAChRs (Stedehouder et al., 2024). The nAChR-dependent increase of DA release is present in non-human primates, although it is smaller in primates than in rodents (Shin et al., 2025).

Evoked dopamine transients by a train stimulation show different patterns of summation depending on recording locations and evoking methods (Rice and Cragg, 2004; Shin et al., 2017; Threlfell et al., 2010; Zhang Y.F. et al., 2025). Dopamine release evoked by stimulation of dopamine neuron terminals show frequency-dependent summation across striatal regions, while dopamine release evoked by ChI stimulation show frequency-dependent summation only in the NAc shell but not in the NAc core or DS (Shin et al., 2017; Figure 1; Table 1). This lack of summation in ChI-evoked dopamine release in the DS and NAc core is caused by either desensitization of nAChRs due to lower activity of acetylcholinesterase or an nAChR-dependent refractory period in the dopamine terminal due to depolarization, which is absent in the NAc shell (Shin et al., 2017). Subunit composition of nAChR on dopamine neuron terminals appears to be different between the DS and VS. Although β2 subunit constitutes presynaptic nAChR in both the DS and VS (Champtiaux et al., 2003; Salminen et al., 2004), the contribution of *α* subunit might be the difference. It is agreed that both α4 and α6 are used in the DS dopamine terminals, while observations in the NAc vary; e.g. using almost exclusively α6 (Exley et al., 2008) or both α4 and α6 (Exley et al., 2011).

Released ACh can modulate dopamine neurotransmission through activation of muscarinic ACh receptors (mAChRs) both in ChIs and dopamine neuron terminals (Threlfell et al., 2010; Shin et al., 2017; Shin et al., 2015; Razidlo et al., 2022). Activation of Gq-coupled muscarinic M5 receptors (M5R) on DA terminals potentiates dopamine transmission, while activation of Gi/o-coupled M2/M4 autoreceptors on ChIs shuts down cholinergic neurotransmission thereby reducing dopamine release (Razidlo et al., 2022; Shin et al., 2017; Shin et al., 2015; Threlfell et al., 2010; Threlfell et al., 2012). Dopamine release modulation through mAChRs is also regionally heterogeneous. Enhancement of frequency dependent dopamine release summation is mediated by both M4R and M2R in the DS, while mostly by M4R in the NAc core and shell (Threlfell et al., 2010; Figure 1; Table 1). Activation of mAChR in ChIs reduces dopamine release evoked by a single stimulus in the DS and NAc; this reduction is also through M2R/M4R (Threlfell et al., 2010). M5R is expressed in fairly limited population of neurons, including ventral midbrain dopamine neurons (Razidlo et al., 2022). In the NAc, activation of M5R, presumably only in dopamine neuron terminals, facilitates dopamine release evoked by optogenetic stimulation of dopamine terminals (Shin et al., 2015; Razidlo et al., 2022). The M5R mediated facilitation is plastic; repeated exposure to stressor reduces M5R mediated dopamine release facilitation without affecting M5R expression level (Razidlo et al., 2022).

ChI-dependent dopamine release can be triggered via glutamate inputs to ChIs (Adrover et al., 2020; Threlfell et al., 2012), suggesting that intrinsic synaptic inputs to ChIs are sufficient to trigger dopamine release as well. In slices, optogenetic stimulation of PFC or thalamic projections in the DS evokes dopamine release, which is blocked by β2*-nAChR antagonists (Adrover et al., 2020; Threlfell et al., 2012). Adrover and colleagues found a “hot spot” within the ventral dorsomedial region of the striatum where optogenetic stimulation of glutamatergic PFC inputs reliably triggered action potentials in ChIs and in turn triggered dopamine release. These excitatory inputs to ChIs were relatively absent in the NAc, despite strong PFC glutamatergic inputs to SPNs (Suska et al., 2013). These observations indicate that dopamine release driven by PFC input through ChI activation is regionally selective; more prominent in the DMS and less so to the NAc or DLS (Adrover et al., 2020).

Dopamine release and its control are different between striosome and matrix as well. Single electrical stimulus evoked dopamine release is larger in matrix than in striosome in the DS (dorsal half of the DS), while dopamine release is smaller in matrix than in striosome in the VS (ventral half of the DS and NAc core) (Salinas et al., 2016). These differences are not due to different contribution of nAChRs to the dopamine transient between striosome and matrix, since antagonist of β2-containing nAChRs suppresses DA release similarly in all compartments (Salinas et al., 2016).

### Heterogeneity in dopamine regulation of ChIs

Meso-striatal dopamine projections are arranged topographically; the most medial VTA dopamine neurons project to ventromedial part of the striatum (the NAc medial shell) and more laterally locating dopamine neurons project to dorsolateral part of the striatum (Haber and Behrens, 2014; Ikemoto, 2007). While midbrain dopamine neurons in both regions share many similar properties, there are significant differences in gene expression, intrinsic excitability, and synaptic connections-- for review see (Basso et al., 2024; Conrad et al., 2024; Fu et al., 2016).

Dopamine reduces ChI firing via dopamine D2 receptors. D2 receptors exert membrane delimited inhibition of voltage gated sodium channels and N-type calcium channels via activation of Gi/o proteins (Maurice et al., 2004; Yan et al., 1997). These mechanisms show onset and offset with order of seconds and can reduce GABAergic and muscarinic PSCs (Pisani et al., 2000). However, electrical train stimulation of thalamic inputs produces subsecond-order D2-dependent pauses following the initial burst in DS ChIs (Ding et al., 2010), and selective stimulation of dopamine neuron terminals also generates D2-mediated subsecond-order firing pause in DS ChIs (Chuhma et al., 2014; Cai and Ford, 2018; Chuhma et al., 2018), which has a similar time course as a pause evoked by salient stimuli *in vivo* (Aosaki et al., 1994a; Kimura et al., 1984). These observations suggest additional faster modes of firing control through D2 receptors. This faster mode of D2 mediated inhibition is due to discrete IPSCs through G-protein coupled inward rectifier K + channels (GIRK) presumably with direct coupling to βγ-subunit of Gi/o. This D2-mediated inhibition through GIRK is not observed in indirect-pathway SPNs despite high expression level of D2R (Chuhma et al., 2023), however, virally-mediated GIRK overexpression renders iSPNs capable of generating D2-IPSCs (Marcott et al., 2014) suggesting strength of D2-IPSC is determined by expression level of effector channels, not D2Rs. The D2-mediated fast inhibition in ChIs is more prominent in the DS than NAc, and stronger in the DMS than DLS (Chuhma et al., 2023; Cai and Ford, 2018; Chuhma et al., 2018; Figure 1; Table 1). Within the NAc, D2-mediated inhibition is observed in the core, while it is substantially weaker in the medial shell (Chuhma et al., 2014; Chuhma et al., 2023; Gallo et al., 2022).

D2R-mediated regulation of ChIs indeed affects behaviors. With selective D2R overexpression in NAc ChIs, mice make more impulsive choices, (i.e., more choices for small immediate rewards than delayed large rewards) (Cavallaro et al., 2023). NAc ChIs in cocaine susceptible mice express more D2Rs compared to control or cocaine resilient mice (Lee et al., 2020). It is possible that upregulating “the pause” in NAc ChIs is responsible for these maladaptive behaviors.

ChIs are a unique population in which the integrative stress response (ISR) pathway is constitutively active. Moreover, the ISR state affects D2-mediated inhibition of ChI firing (Helseth et al., 2021). In the DS, under normal conditions with constitutively active ISR, D2R agonism produces reductions in ChI firing. However, when the ISR pathway is specifically disrupted in DS ChIs, D2R agonism enhances firing through reduction of SK channel activity (Helseth et al., 2021). This ISR disruption enhances stimulus–response and procedural learning behaviors, perhaps making animals more habitual, without affecting motor functions (Helseth et al., 2021).

Selective stimulation of dopamine neuron terminals also causes delayed excitation in ChIs through D1/D5 receptor activation (Chuhma et al., 2018; Wieland et al., 2014). This response was first observed in ChIs in the olfactory tubercles (OT) (Chuhma et al., 2018; Wieland et al., 2014), which is regarded as ventral extension of the NAc shell (Ikemoto, 2007). In contrast to D2R responses, D1/5R responses in ChIs are observed in limited areas in the DLS (Chuhma et al., 2023). While delayed excitation in OT ChIs is solely mediated by D1/5Rs, DLS ChI delayed responses are mediated by D1/5Rs and metabotropic glutamate receptor 1 (mGluR1), sharing Trp3 as the effector channel (Chuhma et al., 2018; Wieland et al., 2014). Latency of the delayed excitation is around 300 ms from stimulus onset; roughly the same as “rebound” firing onset *in vivo* (Aosaki et al., 1994a; Chuhma et al., 2018; Duhne et al., 2024; Wieland et al., 2014; Yamada et al., 2004). With D2R inhibition followed by D1/5R excitation, dopamine neuron inputs can generate pause-rebound response on ChIs in the DLS, although other synaptic inputs are likely to contribute to triphasic response *in vivo*.

### Heterogeneity in glutamatergic and GABAergic neurotransmission onto ChIs from midbrain dopamine neurons

Midbrain dopamine projections co-release glutamate and GABA onto ChIs in a regionally heterogenous manner (Kim et al., 2015; Straub et al., 2014; Stuber et al., 2010; Sulzer et al., 1998; Tecuapetla et al., 2010; Tritsch et al., 2012). Glutamate co-transmission is mediated by vesicular glutamate transporter 2 (*Slc17a6*, vGlut2), and co-expressing dopamine neurons are mostly localized to the medial VTA with a small population in the SNc, projecting to the NAc medial shell and to the anterior DLS, respectively (Cai and Ford, 2018; Chuhma et al., 2014; Chuhma et al., 2018; Poulin et al., 2018). ChIs receive the most prominent glutamate co-transmission, compared to SPNs or fast-spiking GABAergic interneurons (FSIs), but responses are observed only in the medial NAc and anterior DLS (Chuhma et al., 2023; Figure 1; Table 1). Glutamate co-transmission to NAc medial shell ChIs is solely mediated by ionotropic glutamate receptors (iGluRs), and sufficient to cause firing; train stimulation of dopamine neuron terminals, mimicking phasic firing, causes burst firing with very short latency (Chuhma et al., 2014). In DLS ChIs, glutamate co-transmission is mostly mediated through mGluR1 accompanied with small iGluR responses (Cai and Ford, 2018; Chuhma et al., 2018). The mGluR1 response mediates delayed excitation, along with D1/5R response, through activation of Trp3 channels (Cai and Ford, 2018; Chuhma et al., 2018).

In contrast to glutamate co-transmission, GABA co-transmission emanating from dopamine neurons is widely observed across striatal subregions, measured in SPNs and interneurons (Chuhma et al., 2023). Dopamine neuron GABA co-transmission is mediated by two mechanisms; uptake of extracellular GABA and packaging it into vesicles at the terminal via VMAT (Tritsch et al., 2012), or using aldehyde dehydrogenase1a1 for GABA synthesis in dopamine neurons (Kim et al., 2015). GABA co-transmission also contributes to firing pauses in the ChIs, particularly in the NAc core (Chuhma et al., 2014).

By blending dopamine, glutamate, and GABA, dopamine neuron synaptic transmission generates regionally heterogeneous responses in ChIs among striatal subregions. In the DMS, because of absent D1/5R response and mGluR1 responses, dopamine neuron transmission causes a prominent firing pause with subsecond time course via D2R, while it causes a shorter D2R mediated pause and delayed (“rebound”) increased firing due to D1/5R and mGluR1 responses in DLS ChIs (Cai and Ford, 2018; Chuhma et al., 2014; Chuhma et al., 2018; Chuhma et al., 2023). In the medial NAc, including medial core, dopamine neuron input causes immediate burst firing followed by pause due to D2R and SK channel activation (Chuhma et al., 2014; Chuhma et al., 2023). In the lateral NAc, including lateral core, dopamine input generates firing reduction or shorter pause mostly mediated by GABA-A receptors (Chuhma et al., 2014; Chuhma et al., 2023).

### Heterogeneity in VTA GABAergic inputs to ChIs

In addition to meso-accumbal dopamine neurons, we briefly mention long-range GABAergic neurons in the VTA projecting to NAc ChIs with interesting behavioral effects. Using *ex vivo* electrophysiology with optogenetics, it has been demonstrated that these VTA GABAergic projections (VTA^GABA^) form monosynaptic connections on ChIs (Al-Hasani et al., 2021; Brown et al., 2012). They do not form any significant monosynaptic connection to SPNs, suggesting the connection is selective to ChIs (Al-Hasani et al., 2021; Brown et al., 2012). Stimulation of these neurons generates a pause in ChIs and the pause enhances associative learning in a fear-conditioning cue discrimination task (Brown et al., 2012). A subsequent study demonstrated significant regional heterogeneity between the dorsal NAc shell (dNAcSh) and ventral NAc shell (vNAcSh) (Al-Hasani et al., 2021). There is a gradient in strength of VTA^GABA^ input to ChIs; vNAcSh ChIs receive substantially stronger inputs compared to dNAcSh (Al-Hasani et al., 2021).

## Heterogeneity in ChI firing in response to behavioral states

ChIs encode salient stimuli; they respond to novel tones, changes in associative contingencies, or cues for reward delivery (Aosaki et al., 1994b; Apicella et al., 2011; Costa et al., 2025; Duhne et al., 2024; English et al., 2011; Mohebi et al., 2023; Zhang Z. et al., 2025). Classic studies demonstrate that ChIs pause during dopamine neuron burst firing in response to reward delivery—revealing an inverse relationship between ChIs and dopamine neuron firing (Aosaki et al., 1994b; Bromberg-Martin and Hikosaka, 2009; Shimo and Hikosaka, 2001). The ChI pause during associative learning is thought to be a permissive temporal window for dopamine-dependent plasticity by suspending ACh transmission (Calabresi et al., 2007).

The characteristic burst-pause-rebound response of TANs acquired through learning (Aosaki et al., 1994a; Aosaki et al., 1995), in a dopamine-dependent manner (Aosaki et al., 1994a), can be observed across different striatal subregions. Nevertheless, there are some variations depending on the subregions and behavioral contexts. For example, modulation of the pause phase does not occur uniformly across striatal subregions; firing in DMS ChIs varies more and is closely tied to learning phases than in DLS ChIs (Thorn and Graybiel, 2014). Following extinction or changes in task contingencies, the pause response can diminish or transform (Matsumoto et al., 2001; Ravel et al., 2001).

**Figure 2:**
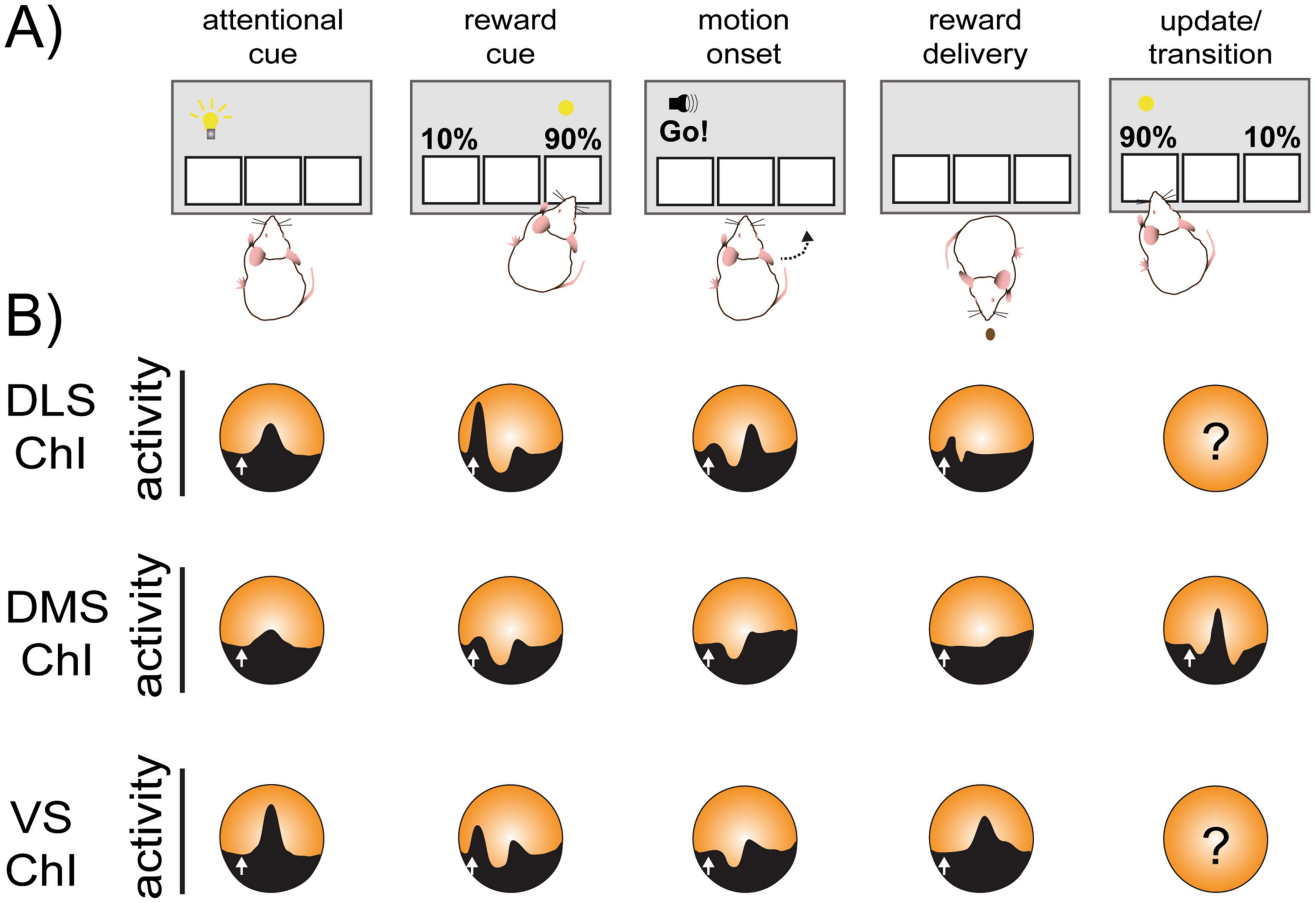
Summary of ChI activity pattern heterogeneity *in vivo* during reward-guided behavior. **(A)** Cartoon depicting a generic operant procedure performed in rodents in which neural responses to specific task elements such as movement onset, reward-predictive cue delivery, reward delivery, and others can be assayed. **(B)** Typical *in vivo* ChI activity recorded electrophysiologically or with calcium indicator across striatal subregions in response to different tasks. Question marks in the circle indicate that data has not been reported. White arrows indicate the stimulus onset.

Duhne et al. (2024) conducted recording from DLS, DMS and VS (mainly, NAc core) ChIs in rats during an adaptive decision-making task with probabilistic reward (two-armed bandit task) (Hamid et al., 2016; Figure 2A). This task allows comparison of ChI activity across subregions during different behavioral states, namely, motivational state, decision making, movement execution, reward expectation and reward delivery. In response to an unexpected reward, DLS ChIs exhibited a classic burst-pause-rebound response. DMS ChIs did as well, however, the burst response was significantly smaller. In contrast, VS ChIs showed an increase in activity on average, although responses were much more heterogeneous. While ChIs in all recorded regions responded to trial start cue (light on), execution of movement cue (“Go! Cue”), and reward delivery, these ChI responses were heterogeneous among subregions (Figure 2B). The activity of VS ChI was more affected by motivation and reward states compared to DS ChIs. At the initial phase of two-armed bandit task, animals are expected to approach and poke a central port after trial start cue (port light on). The latency from the cue to nose poke reflects motivation to perform the task; the shorter latency is regarded as a more “engaged” motivational state (Hamid et al., 2016). VS ChIs showed the largest response to trial start cue in motivated trials (latency < 1 s), compared to DLS or DMS ChIs (Duhne et al., 2024). Moreover, a reward prediction error (RPE) encoding via firing increase during reward delivery was observed only in VS ChIs, suggesting that VS ChI activity is related to motivation and reward processing. In the same study, in response to the Go! Cue, DLS ChIs showed a burst-pause-rebound triphasic response, while DMS ChIs showed only a pause. VS ChIs showed slower increase in firing with a less defined pause (Figure 2B). In primates, DMS (caudate) TANs respond more to instruction signal associated with reward, while DLS (putamen) TANs respond more to cue for movement initiation, although the response types are not totally segregated by location (Yamada et al., 2004). Similarly, TANs in both DLS and VS respond around movement initiation and execution with direction selectivity (firing is different by choosing ipsilateral or contralateral side of recording), though DLS TANs direction selectivity was greater than VS TANs (Yarom and Cohen, 2011).

Though not a direct measure of ChI firing, a recent study assayed ACh release across multiple locations, measured by G protein-coupled receptor Activation-Based ACh sensor (GRAB-ACh3.0; AAV9-hSyn-ACh3.0, WZ Biosciences). Different locations show a substantial diversity in responses to unexpected reward, even closely located sites demonstrates a distinctive response profile (Bouabid et al., 2025). Conditioned stimuli or reward delivery evokes triphasic (peak-dip-peak) ACh release, although not all recorded locations show all three phases. During pavlovian conditioning and its extinction, both positive and negative RPE are encoded in distinct phases of ACh release. They found positive RPE is encoded as initial ACh peaks in the VS and as ACh dips in the anterior DS. In contrast, negative RPE is encoded as “the pause” following ACh release, supporting the hypothesis that ChIs provide behavioral flexibility, that is to transition from one behavioral state to another (Amaya and Smith, 2021; Bouabid et al., 2025; Bradfield et al., 2013; Gangal et al., 2023; Groman et al., 2011; Huang et al., 2024; Okada et al., 2014; Poppi et al., 2021).

When ChIs are recorded in the DMS during tasks that require updating action-outcome association, disruption of the pauses with optogenetic stimulation impairs the update, suggesting a role of the pause in behavioral flexibility (Huang et al., 2024). In previous literature behavioral flexibility has been mostly associated with ChI activity in the DMS, as expected from roles of the DMS in goal-directed behavior. However, ChIs in the DLS or NAc are also indicated to be involved in flexibility in behavioral repertoires in some studies (Amaya and Smith, 2021; Aoki et al., 2018). This is consistent with negative RPE during extinction represented in some lateral DS regions (Bouabid et al., 2025).

Considering substantial difference in responses by recording location, some of the discrepancies across studies may be due to spatial specificity. Another possible factor is in measurement methods with different spatial and temporal resolution. While electrophysiological recordings have sub millisecond temporal resolution, genetic sensors capture population-averaged signals with temporal resolution on the order of 10–100 s of milliseconds. Additionally, electrophysiological methods or genetic Ca^2+^ indicators in ChIs indirectly measure ChI activity, while genetic ACh indicators measure ACh transmission (dependent on GPCR binding and conformational changes that produce fluorescence from post-synaptic cells) as a proxy of release. If ACh release does not increase linearly with ChI firing, there might be local modulation at ACh release site. Thus, ChI activity measurements and ACh measurements may produce different peri-event profiles.

When ChI activity and ACh release were recorded in parallel experiments during Pavlovian or operant conditioning tasks, both ChI activity and ACh neurotransmission increase with reward-predictive cues and decrease at reward delivery by progression of conditioning in the ventral NAc shell, while these are not observed in the dorsal NAc shell (Al-Hasani et al., 2021). Stimulation of VTA GABA projection silences ChI activity in ventral NAc shell and enhances real-time place preference, while stimulation of VTA GABA projection in dNAc shell or NAc core does not have effects in these reward-guided tasks (Al-Hasani et al., 2021). This study is a good example of the utility of measuring both ChI activity and ACh neurotransmission patterns to assess possible cellular mechanism. These observations suggest that finer regional heterogeneity of synaptic transmission on ChIs indeed mediates discrete behaviors even within what could be broadly defined as the NAc or even NAc shell.

## Heterogeneity in ChI-dopamine interaction across striatal regions *in vivo*

Some of the first evidence of nAChR-dependent elevations of dopamine in the striatum *in vivo* came from the addiction field, showing that acute administration of nicotine elevated dopamine levels in the DS and VS over minute-long timescales in rodents using microdialysis (Nakamura et al., 1992; Toth et al., 1992). *In vivo* corroboration of the precise reciprocal interactions between ChIs and dopamine varicosities and relevance to observations *ex vivo* have historically been sparse and inconsistent. However, very recent work has illuminated the conditions for successful obervation of the ChI-dopamine interaction *in vivo*.

*In vivo* simultaneous measurement of ChI activity with genetic Ca^2+^ indicator (GCaMP6f) and dopamine release with genetic dopamine indicator (RLight) revealed that activation of ChIs and dopamine release occur at about the same time in the NAc core in response to reward-prediction cues. During motivated approach in an operant task, both ChI activity and dopamine release show a ramping profile (Mohebi et al., 2023). While dopamine release and ChI firing at reward-prediction cue are accompanied by VTA dopamine neuron phasic firing, ramping of dopamine release and ChI activity during motivated approach is independent from VTA dopamine neuron activity, suggesting the ramping of dopamine release is due to local control through ChIs (Mohebi et al., 2023). However, in later work monitoring ACh and dopamine neurotransmission simultaneously in the NAc core, ACh does not ramp coincidently with dopamine ramps during motivational approach, while task-starting cue causes increase of dopamine and small increase of ACh followed by a “dip” (Costa et al., 2025).

Duhne and colleagues examined timing of dopamine release and ChI firing pause in the DMS, DLS, and VS (NAc core) following a movement-execution cue signal (Go! Cue). Both dopamine release and the firing pause are cue-locked in all locations, but the pause initiates slightly before dopamine release. In contrast, the “rebound” increase of firing in DLS ChIs is movement-locked and follows dopamine release. The reward cue-associated phasic dopamine release scaled in accordance with reward prediction error in all three recorded regions, while only VS ChIs showed this RPE-scaled increase in firing (Duhne et al., 2024). This indicates a more predictable ChI→ACh→dopamine sequence of cellular events in the NAc during reward processing than in the DS, despite these systems being engaged by the task in both regions. Simultaneous recording of dopamine and ACh release from DLS during a similar probablistic decision-making task showed that dopamine release was independent from ChI-mediated nAChR activation, although dopamine and ACh release are strongly affected by decision history and reward outcomes (Chantranupong et al., 2023).

Simultaneously measured dopamine and ACh (or ChI activity) are constantly oscillating in the DS (Krok et al., 2023), and in the NAc core (Mohebi et al., 2023). In the DLS, dopamine and ACh oscillations show an overall 90° phase shift (digitized at 5 kHz); ACh “dip” is delayed from dopamine peak about by 120 ms (Krok et al., 2023) indicating anticorrelation. This “delay” of ACh dip is also observed in the NAc core at task starting cue (Costa et al., 2025). The phase shift is observed regardless of the behavioral status, i.e., during locomotion, motionless, or with reward delivery (Krok et al., 2023). The same pattern of oscillation is observed also in the DMS, although responses in two striatal subregions are not coherent (Krok et al., 2023).

Touponse et al. (2026) examined coincident ACh and DA neurotransmission in the anterior dorsomedial NAc core of mice in an operant conditioning task with high and low effort requirement (Touponse et al., 2026). Using a multidisciplinary approach, the investigators dissociated ChI-nAChR dependent dopamine transmission in the NAc from dopamine transmission solely dependent on somatic firing. They concluded that ChI-dependent dopamine release was recruited under conditions of high, but NOT low effort requirement. In *ex vivo* conditions, there is a minimum threshold of synchronous activity of ChIs to evoke detectable a dopamine signal using electrochemical methods as well as photometry. Perhaps this study provides insights into the behavioral conditions, i.e., high effort requirement) necessary to synchronize a minimum number of ChIs to cause dopamine release.

Goldbach et al. (2025) found that different cue modalities differentially recruited ChI-triggered dopamine transmission in the DMS. Specifically, visual, but not auditory stimuli caused ChI-dependent dopamine release driven by projection from the prefrontal cortex, suggesting different sensory inputs trigger different modulatory mechanisms on ChI-dopamine interaction (Goldbach et al., 2025). This observation is another example of task-dependent recruitment of ChI-driven dopamine release.

Thus, discrepancies among these observations, for example, whether or not the cue signal causes coincident increase of dopamine and ChI activity, may be due to recording location or behavioral task. A third factor to be considered is temporal resolution as mentioned in a previous section. A study by Flink et al. (2025) demonstrated that when dopamine release is measured with dLight1.2 in the DS through cranial windows with very fast sampling rate (500 frame-per-second; fps), distinct ChI-driven dopamine peaks were observed with a nAChR-mediated fast peak and a mAChR-mediate slow-peak, While 16 fps sampling rate obscure the nAChR-mediate fast-kinetic peak (Flink et al., 2025). These data point to the importance of temporal resolution to assess interaction between ACh and dopamine properly *in vivo*. Combination of genetic/viral tools with higher spatiotemporal resolution and regionally precise recording might help to study distribution of strength of ACh-induced dopamine release *in vivo*.

## Regional heterogeneity in contribution of ChIs to different basal ganglia associated disorders

ChI dysfunction has been linked to neuropsychiatric disorders, including Parkinson’s disease (PD), Hungtington’s disease, substance abuse and depression. In Parkinson’s disease, elevations of ACh neurotransmission occurs at the same time as dopamine cell death (Aosaki et al., 2010; Lester et al., 2010; Sanchez-Catasus et al., 2022). Therefore, patients were historically prescribed anticholinergic drugs alongside L-DOPA. However, recent preclinical work shows a potentially contradictory observation; in PD model mice, increasing ACh selectively in the DLS enhances the L-DOPA effect, thereby increasing dopamine release, and rescuing the movement deficit (Li et al., 2024). The authors argue that local ACh modulation and systemic ACh modulation might have different results, which again corroborates the notion of striatal region-specific control of dopamine/ACh modulation. In the same study, L-DOPA mediated increase of dopamine release and decrease of ACh are observed only in PD model mice, not in healthy controls, suggesting that the PD condition perturbs mechanisms of reciprocal modulation between dopamine and ACh, which contributes to deficits in movement (Li et al., 2024). Likewise, in the DMS, disruptions in the reciprocal relationship between dopamine neurons and ChIs can cause perseveration and inflexibility (Bradfield et al., 2013; Okada et al., 2014). Furthermore, in the NAc reductions in ChI firing or ACh release can lead to depressive-like phenotypes and addiction-like phenotypes (Abudukeyoumu et al., 2019; Cavallaro et al., 2023; Cheng et al., 2019; Gallo et al., 2022; Lee et al., 2020; Warner-Schmidt et al., 2012). Reduction of ChI firing using genetic modification and Gi-DREADD activation promotes depressive-like phenotypes in mice. These NAc ChI specific manipulations reduce evoked dopamine release in the NAc in response to appetitive stimuli (Cheng et al., 2019; Hanada et al., 2018; Warner-Schmidt et al., 2012), again supporting the critical ACh-dopamine interaction in adaptive behaviors and positive functional correlation.

Heterogeneous intrinsic properties of ChIs may also contribute to differential susceptibility to pathological conditions. In PD, SNc dopamine neurons degenerate while VTA dopamine neurons are relatively spared (Brichta and Greengard, 2014). This is likely due to differences in Ca^2+^ channel activity and relative Ca^2+^ demand between SNc and VTA dopamine neurons, which activates different mitochondrial signaling and in turn makes SNc neurons more susceptible to oxidative stress (Surmeier, 2007; Surmeier et al., 2017). We speculate that the combination of different intrinsic properties and their modulation could make ChIs in different striatal subregions differentially sensitive to pathological conditions, as observed in dopamine neurons. For example, we observed that NAc core and NAc shell ChIs are differentially affected by repeated stressor exposure, and this impacts their sensitivity to corticotropin releasing factor (Ingebretson et al., 2024). This might be related to differential firing frequencies and synaptic modulations in the NAc.

## Concluding remarks

Striatal ChIs have been the center of several studies due to their prominent control of striatal circuits, unique connectivity with dopamine neurons and diverse responses during behavioral tasks. Recent studies have featured regional heterogeneity in ChI activity and its modulation, particularly reciprocal interactions between dopamine and ACh at different scales within the circuit. *Ex vivo* electrophysiology offers fine analysis of intrinsic and synaptic mechanisms by isolating distinct cells and synapses, while *in vivo* physiology reveals how these signals are combined to shape neural circuits in intact conditions. Anatomical mapping defines organizational principles, and behavioral assays establish relevance of neuronal activities to adaptive and maladaptive states. All these experimental results point to very precise spatial and temporal modulation of ChI activity. However, still there is a gap between *ex vivo* heterogeneity studies and *in vivo* observations. Future studies are required to fill the gap, presumably using high resolution recordings with precisely timed and/or localized manipulations *in vivo* or, for example, by using precise genetic manipulations to target specific ACh receptors in dopamine neurons (i.e., β2-containing nAChRs from VTA dopamine neurons). We have summarized these conclusions in Box 1. To draw an entire picture of ChI heterogeneity and its functional relevance, distinct experimental preparations cannot be left behind, and deeper integration across levels is essential. Cross-disciplinary collaboration is necessary to ensure this research coalesces and progresses.

### Future directions and suggestions

We have identified three main sources of the inconsistencies found both across *in vivo* studies and when comparing *in vivo* and *ex vivo* studies. These are (a) spatial resolution, (b) temporal resolution, and (c) task parameters or behavioral contexts. Three future experimental advances that could resolve these issues are as follows:

- Incorporate use of high-density neural recordings in combination with optogenetics in order to record from a larger number of ChIs over different subregions of the striatum to achieve greater spatial and temporal resolution.
- Determine how the triphasic response of ChIs is shaped *in vivo*. *Ex vivo* studies indicate multiple candidates of each component of the triphasic responses. However, *in vivo* responses differ by subregions and dynamically change with behavioral status. Now that we have established diversity of “pause” mechanisms for example, we can utilize a combination of viral and transgenic strategies to determine which mechanisms are engaged under different behavioral conditions.
- Use computational modeling and simulation combined with *in vivo* single cell electrophysiology to determine how intrinsic excitability determines the impact of different synaptic inputs.
